# The Age-Related Efficacy of Dimethyl Fumarate in Naive Versus Switcher Multiple Sclerosis Patients: A Multicenter Population-Based Study

**DOI:** 10.3390/ph18111730

**Published:** 2025-11-14

**Authors:** Roberto De Masi, Stefania Orlando, Assunta Greco, Maria Carmela Costa

**Affiliations:** 1 Laboratory of Neuroproteomics, Multiple Sclerosis Centre, “F. Ferrari” Hospital, 73042 Casarano, Lecce, Italy; 2 Complex Operative Unit of Neurology, “F. Ferrari” Hospital, 73042 Casarano, Lecce, Italy; 3 Departmental-Level Simple Operative Unit of Cardiology, “F. Ferrari” Hospital, 73042 Casarano, Lecce, Italy; 4 Complex Operative Unit of Ophthalmology, “V. Fazzi” Hospital, 73100 Lecce, Italy

**Keywords:** dimethyl fumarate, naive patients, drug efficacy, multiple sclerosis, treatment switchers

## Abstract

**Background:** The role of age and time-dependent variables in determining the response to disease-modifying therapies (DMTs) has aroused growing interest in the Multiple Sclerosis (MS) field. Although it is a very hot topic, related literature on the subject is considerably lacking. Objectives: The aim of this study is to deepen the understanding of how time-dependent variables influence disability accumulation and drug response in an MS population, assuming DMF as the first-line treatment, and to expand our knowledge of the risk–benefit evaluation of DMF. **Methods:** We investigated, in a real-world setting, the efficacy of Dimethyl Fumarate (DMF) in naive versus switcher MS patients, correlated with age, in preventing disability accumulation. Starting from an initial population of 234 DMF-treated patients, we selected 169 of them based on their similar time in therapy (TinT) with DMF of 5.9 ± 2.3 year and sex ratio. Of these, 74 were naive and 95 were lateral switchers at the start of treatment. The mean Expanded Disability Status Scale (EDSS), Disease Duration (DD), age and age at onset were compared between groups. **Results:** The switcher group showed higher EDSS and age compared to the naive group (2.7 vs. 1.8, *p* < 0.001; 40.2 vs. 35.5, *p* = 0.005, respectively). Age correlated with DD, EDSS and age at onset in both naive (r = 0.39, *p* = 0.007; r = 0.53, *p* = 0.000; r = 0.63, *p* = 0.000, respectively) and switcher (r = 0.46, *p* = 0.002; r = 0.49, *p* = 0.000; r = 0.61, *p* = 0.000, respectively) groups. Kaplan–Meier curves, adjusted for age, also indicated that the naive group retained an EDSS score status of 0.5–3.5 more frequently (*p* < 0.001) and reached elevated disability less frequently (*p* = 0.002) than switchers. The mean EDSS percentage ratio between paired naive and switcher patients, representing the differential neurological impairment (DNI), was 69%, inversely correlating with age in both naive (r = −0.52, *p* < 0.001) and switcher patients (r = −0.47, *p* < 0.001). Finally, logistic regression analysis indicated age as an independent and predictive variable with respect to EDSS. **Conclusions:** We conclude that age is the main contributor to disability progression and the primary predictive factor for treatment effectiveness for DMF in both naive and switcher MS patients.

## 1. Introduction

Multiple sclerosis (MS) is a chronic disorder of the central nervous system (CNS) with an inflammatory and degenerative component, representing the first cause of juvenile non-traumatic disability, with an estimated incidence of 30/100,000 cases in the general population [1]. This pathology is characterized by auto-immune lymphocytic infiltration of the blood–brain barrier (BBB), resulting in inflammatory demyelination and axonal transection. These processes are classically considered focal or multifocal in nature, although a diffuse involvement of the normally appearing white matter and grey matter was immunohistochemically demonstrated by Trapp in 1999, using SMI 32 antibodies [2]. Notwithstanding the still unknown etiology, it is a common opinion that cellular immunology of MS recognizes molecular mimicry as the pathological *primum movens*. Successively, the peripheral lymphocyte is activated, followed by epitope spreading with BBB disruption; CNS invasion; local reactivation in a context of tissue damage; and, finally, process perpetuation, resulting in subsequent inflammatory compartmentalization [3] and the formation of subpial ectopic lymphoid follicles [4]. After a first demyelinating attack, indicated as clinically isolated syndrome, these events often evolve into recurrent episodes of neurological dysfunction, after which patients experience partial or complete clinical improvement, according to the relapsing-remitting disease course of MS (RRMS). In the later stage of RRMS, axonal loss becomes prevalent and exceeds the neurological reserve, finally leading to a gradual reduction in functional recovery and silent progression of disability [5]. Already detectable in the early pathological stages, the silent progression is considered the main pathophysiological contributor of the transition phase, which, in turn, leads to the secondary progressive phenotype of MS (SPMS) [6]. In fact, the primary driver of MS is considered the neuroinflammation of smoldering lesions, which ultimately leads to the loss of oligodendrocytes and neurons, according to a biological *continuum* of the disease [7]. Despite its worldwide use, the current MS classification is criticized because it is based on subtypes that apparently belong to a disease *continuum* rather than distinct biological entities. In the primary progressive form of the disease, neurodegeneration is evident *ab initio*, with an inflammatory phase considered absent or early transient [8]. This difference in underlying mechanisms explains why traditional disease-modifying therapies (DMTs) are anti-inflammatory in nature, being effective in RRMS forms but often ineffective in progressive forms.

Therefore, it is believed that early initiation of DMTs may prevent neurological damage, acting on neuroinflammation and smoldering lesions formation, ultimately preventing disability accumulation and disease progression [9,10]. Obviously, neurological reserve is limited and inherently age-dependent, limiting functional recovery and restoration after inflammatory demyelination, ultimately leading to disability accumulation and clinical progression [11,12]. Telomere shortening and damage are recognized causes of cellular senescence and immune aging in MS, causing a chronic inflammatory state, as well as reduced safety and efficacy of DMTs [13].

In this context, Dimethyl Fumarate (DMF) exerts immunomodulatory properties mediated by the activation of nuclear factor (erythroid-derived 2)-like 2 (Nrf2) and hydroxycarboxylic acid receptor 2 (HCAR2) with a detectable anti-inflammatory and cytoprotective effect [14,15]. Furthermore, given its protection against experimental diabetes due to its anti-proteotoxic effect, DMF could also affect the neurological misfolding process, as recently suggested by our research group, as a pathological contributor in MS [16].

Two multinational, double-blind, placebo-controlled, Phase 3 studies (DEFINE and CONFIRM) evaluated the efficacy and safety of DMF versus placebo and demonstrated a significant reduction in the relapse rate (RR) and the proportion of confirmed disability progression and MRI parameters, including post-contrast T1-weighted and T2-weighted lesion load (LL), in the recipients [17,18]. The five-year extension of DEFINE/CONFIRM, called the ENDORSE study, also showed a reasonable safety profile of the molecule, expressing a similar incidence of adverse events (AEs) and correlated discontinuations between patients from the DMF arm and those from the placebo arm [19]. However, these studies were conducted, as expected for all randomized, controlled clinical trials, regardless of patient subpopulations, in a wide and unselected MS population with an age range of 18 to 55 and the diagnosis of relapsing–remitting MS defined in accordance with the 2017 McDonald criteria [20]. Since then, several meta-analyses have been performed, and different patient subpopulations have also been evaluated, including both naive and early-stage MS patients [21,22,23]. However, these studies are limited to the mere measurement of RR and MRI parameters of efficacy, regardless of the physiopathological role of the involved clinical variables, such as age, disease duration, persistence in therapy and age at onset, the so-called time-dependent clinical variables.

Nonetheless, the assessment of patient subpopulations is crucial to consider their different, often particular immunological constitution, which, in turn, determines the response to DMT. In the case of the treatment of naive MS patients, a lot of literature has investigated the immunosenescence phenomenon [24,25,26,27]. However, its association with aging and the effects on diseases have not been clearly revealed, especially in association with a given DMT and the individual response to it. In fact, due to extreme intra- and inter-individual immunological variability, as well as the incomplete representativeness of the circulating cell pool (2% of the total), precision medicine has largely failed in the field of MS with regard to both patient subgroups and the single component. This constitutes a negative turning point, since the regulating law of the drug response cannot be applied to the single component unless first inferred from the belonging group of disease.

Recently, our group and the Weideman group [10,28] have dealt with the topic of how time-dependent variables influence drug response, as well as disability progression, pointing to age as the main contributor to DMT response in MS. In our previous study, we noted reduced effectiveness of DMT at ages of 38–40 years in MS patients, also expressing EDSS ≥ 3.5 [10]. However, how chronological age modifies drug response and disability in the MS population has never been studied using DMF as the first-line treatment. Indeed, this is precisely the aim of this study, which does not represent a sterile academic effort but a further clinical attempt to expand our knowledge of the MS pathology, as well as the risk–benefit evaluation of DMF.

## 2. Results

### 2.1. Data from Descriptive Statistics

We considered two DMF study groups: 74 naive patients and 95 lateral switchers at the time of drug initiation. Each patient was assessed once at enrollment. All enrollments occurred over the three-year study period, and relative data were collected cross-sectionally. The average time in therapy (TinT) with DMF value was similar between the naive and switcher groups: 5.77 ± 2.13 and 6.14 ± 2.54, with no statistical difference between means (*p* = 0.432). In contrast, the mean age was 35.5 ± 10.3 and 40.2 ± 8.7 years, and the mean disease duration (DD) was 5.9 ± 2.7 and 11.2 ± 7.1 years for naive and switcher groups, respectively, with significant differences between means (*p* = 0.005 and *p* < 0.001, respectively). In fact, due to the time spent on other therapies, switchers have a higher DD than naive patients. The mean age at onset was very similar between the two groups: 29.0 ± 5.4 and 29.6 ± 4.5 for naive patients and switchers, respectively, with no statistical difference (*p* = 0.566). The mean Expanded Disability Status Scale (EDSS) was 1.88 in the naive group and 2.72 in the switcher group, with statistical difference (*p* < 0.001). This differential neurological impairment (DNI) represents the mean measure of disability excess in the switcher group compared to the naive group, resulting in a mean of 69%. The naive group showed a sex ratio of 1.9:1.2, while the sex ratio of the switchers was 1.8:1.1, both favoring women, with no significant difference in gender proportions (χ^2^ = 0.413). The two study populations expressed a statistically different Annual Relapse Rate (ARR) in the year before the start of DMF (ARR_pre_): 0.87 ± 0.56 and 0.48 ± 0.28 in the naive and switcher groups, respectively (*p* < 0.001). We also noted a significant difference regarding the ARR in the year after the start of DMF (ARR_post_) between groups: 0.01 and 0.11 in the naive and switcher groups, respectively (*p* = 0.015). The demographic data, along with measures of mean and standard deviation of examined variables, are summarized in Table 1. According to the exclusion criteria, 65 patients were not considered (27.8% of the total).

### 2.2. Data from Inferential Statistics

We found that age correlated with DD, EDSS and age at onset in both naive patients (r = 0.39, *p* = 0.007; r = 0.53, *p* = 0.000; r = 0.63, *p* = 0.000, respectively) and switchers (r = 0.46, *p* = 0.002; r = 0.49, *p* = 0.000; r = 0.61, *p* = 0.000, respectively). Furthermore, EDSS correlated with DD and age at onset in both naive (r = 0.38, *p* = 0.027; r = 0.18, *p* = 0.025, respectively) and switchers (r = 0.35, *p* = 0.01; r = 0.16, *p* = 0.027).

**Figure 1:**
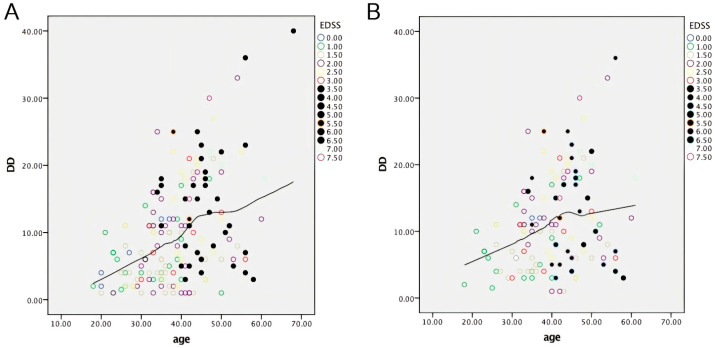
Scatter plots illustrating the correlation between age (years) and DD (years) with EDSS-labeled cases from the naive (**A**) and switcher (**B**) MS groups. EDSS cases with scores of 3.5 to 6.5, seen in bold, show a marked slope curve in both groups within the age range of 35–40. EDSS—Expanded Disability Status Scale.

As shown in Figure 1, the correlation between age and DD with EDSS-labeled cases from the two study groups highlights that patients with higher EDSS scores clustered beyond an ideal vertical cut-off of 39–40 years, supporting the existence of an age-related threshold for reaching EDSS 4.0.

**Figure 2:**
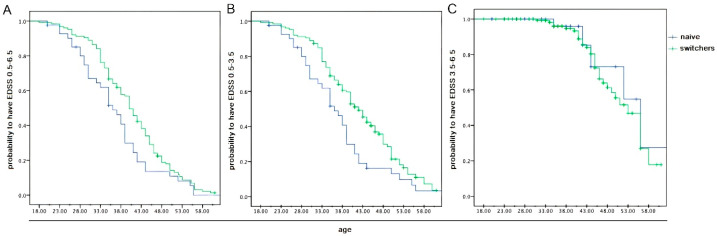
(**A**) Kaplan–Meier curves showing the distribution of patients reaching an EDSS range of 0.5–6.5. The lower curve represents the smaller proportion of naive patients who reach elevated disability, maintaining a low degree of neurological impairment (*p* = 0.002). (**B**,**C**) Kaplan–Meier curves showing the distribution of patients reaching EDSS sub-ranges of 0.5–3.5 and 3.5–6.5 compared to age, reaching significance in the first case and remaining non-significant in the second (*p* < 0.001, *p* = 0.714, respectively). EDSS—Expanded Disability Status Scale.

We also used Kaplan–Meier curves to evaluate patients who achieved an EDSS score status of 0.5–6.5 in both the naive and switcher MS groups. Specifically, in paired patients belonging to the two compared groups, the curve returns the estimated risk of disability at the same age. Figure 2A shows the significant inter-group difference that was found (*p* = 0.002). Stratification by EDSS ranges of 0.5– 3.5 and 3.5–6.5, applying the same statistical analysis, produced divergent results: significance was found in the first case (*p* < 0.001), while no significance was observed in the second (*p* = 0.714), as illustrated in Figure 2B,C.

**Figure 3:**
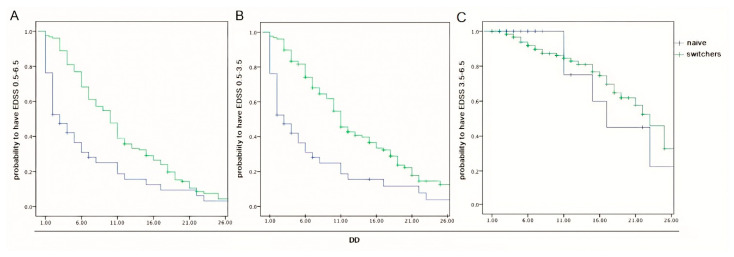
(**A**) Kaplan–Meier curves showing the distribution of patients reaching an EDSS score of 0.5–6.5, (**B**) 0.5–3.5 and (**C**) 3.5–6.5 compared to the DD. Note: In (**A**,**B**) the lower curve represents the smaller proportion of naive patients reaching an EDSS score of 0.5–6.5 and 0.5–3.5 (*p* = 0.017, *p* < 0.001, respectively). No correlation was found in the EDSS score range of 3.5–6.5 (**C**) between naive and switcher groups (*p* = 0.331). DD—disease duration; EDSS—Expanded Disability Status Scale.

We assessed the same milestones compared to DD. Also in this case, Kaplan–Meier curves were suggestive, highlighting a strong correlation of DD with disability, reaching EDSS 0.5–6.5 (*p* = 0.017) and 0.5–3.5 (*p* < 0.001), respectively. Specifically, in paired patients belonging to the two groups, the curve returns the estimated risk of disability at the same DD. No correlation was found in the EDSS score range of 3.5–6.5 (*p* = 0.331). This analysis can be seen in Figure 3.

**Figure 4:**
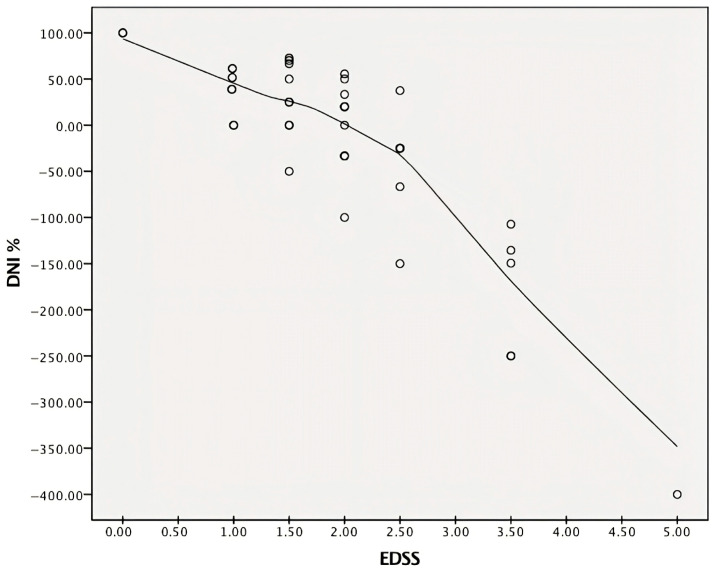
Correlation between DNI and EDSS scores for both naive and switcher patients. A decline in DNI values is observed from lower to higher levels of disability, suggesting that the largest difference between naive patients and switchers occurred at lower EDSS levels. DNI—differential neurological impairment; EDSS—Expanded Disability Status Scale.

We found that DNI values of the switcher group increased from −350.0% to 100.0% from age 18 to 65, associated with an EDSS range of 2.0–6.5 (Figure 4).

**Figure 5:**
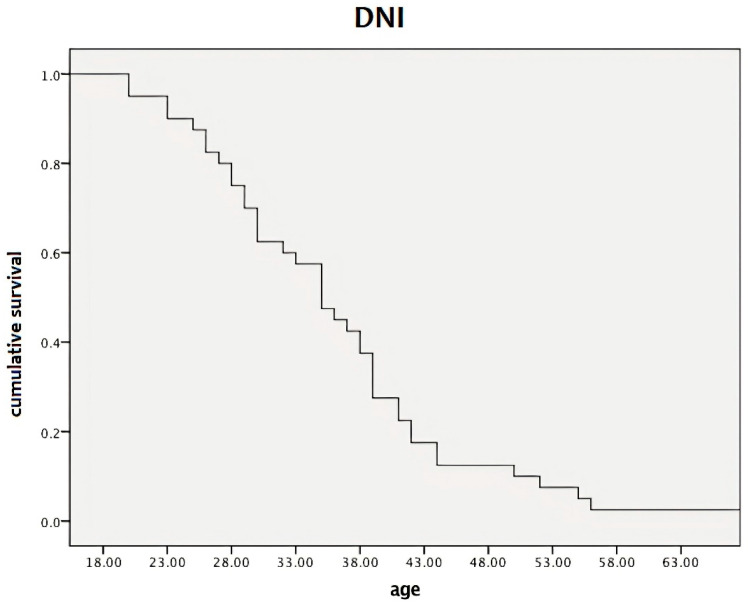
Kaplan–Meier curves of DNI for both naive and switcher patients across the evaluated age range of 18–65 years. The observed constantly sloped curve underscores the rapid age-dependent convergence of disability outcomes between naive and switcher groups. After age 43, the slope curve reduces, indicating a plateau effect. DNI—differential neurological impairment.

The mean DNI value was 69%, with a 95% CI of 52.7% to 10.8%. Figure 5 shows the Kaplan–Meier curve of DNI for both naive and switcher patients across the evaluated age range of 18–65 years.

Finally, it was found that DNI was inversely and significantly correlated with age and EDSS in both naïve patients (r = −0.52, *p* < 0.001; r = −0.7, *p* < 0.001, respectively) and switchers (r = −0.47, *p* < 0.001; r = −0.76, *p* < 0.001, respectively). We observed only a trend between DNI and DD in both groups (specifically, r = −0.24, *p* = 0.082 for the naive patients and r = −0.29, *p* = 0.086 for switchers).

A total of 10 (5.9%) of the studied patients manifested disease activity, 9 of whom (90%) were switchers. The mean age of the switchers manifesting disease activity was 40.4 ± 3.1 years, with an EDSS of 5.5 ± 2.1 and DD of 11.1 ± 3.4. A large proportion of these patients—four patients, 48.4%—worsened clinically, meeting confirmed disability progression (CDP) criteria, without a detectable increase in LL. In fact, only in three patients (28%) expressing disease activity did we find new MRI abnormalities with increased LL.

## 3. Discussion

The meaning of positive correlations among time-dependent variables and between these and disability is not merely a statistical exercise but a description of how neurological impairment accumulates, influenced by neurosenescence and immunosenescence, as well as by the response to DMT in MS. In the present work, these correlations are consistent with the pathophysiology of the disease and are confirmed independently in both switcher and naive populations. Similar correlations have also been found in our previous studies [10].

In fact, the role of age and time-dependent variables in determining the response to DMTs has aroused growing interest in the MS field. For this purpose, not only DMF but also Siponimod and the β-1a Interferon have been studied. At present, the correlation between these variables and the real-world effectiveness of DMT in clinical practice remains undefined. In the recent past, our group and, previously, the Weideman group have focused on this field [10,28,29,30]. However, the latter used a statistical variable not applicable here, since it refers to the placebo arm, which is absent in the present work. A similar concept concerning the differential evaluation of disability between compared groups has been assessed throughout the DNI. DNI has previously been detailed elsewhere [10]. In the present work, it represented the proportion of difference ratio in EDSS scores between individual naive patients and their paired switchers, despite any possible mean age difference between members of the comparison groups. In fact, the higher mean age of the switchers could be considered a confounding factor, given that older people and prolonged DD are naturally associated with an increased likelihood of disability. However, here, the Kaplan–Meier curves and DNI were calculated, matching each patient belonging to the two compared groups by age, finally obtaining the estimate of the risk of disability at the same DD and age values.

During a three-year retrospective, cross-sectional, clinical MRI study, we evaluated, in a population-based setting, the statistical correlation between age, age at onset, DD and disability accumulation in two groups of patients: patients naive at DMF initiation and switchers from other treatments. Based on the patient selection, this study has the merit of evaluating an MS population that is homogenous for TinT with DMF. This allowed us to assess age as a contributor, as well as a predictive variable, in disability progression. Furthermore, the comparison of two different patient populations clarifies how the drug response adapts to their biological conditions, which are already different in nature at the time of clinical observation.

It is known that naive MS patients present immunological and phenotypic characteristics that are different from those of patients who have already received therapy and are prone to undergoing profound and not yet known modifications of the immune system over time. Although naive MS patients present with lower age than other MS subpopulations, this difference was minimized here, although also noted. Specifically, it was minimized by the application of DNI, avoiding senescence bias in the comparison between paired subjects, and noted by comparing two populations that express different mean ages through the application of Kaplan–Meyer curves. Unlike their classical use, which is prospective in the literature, the curves presented here in cross-sectionally compare the EDSS from the individual matched patients, regardless of the mean value of the belonging group. In this way, we achieved several findings. In fact, our naive patients have a mean age, DD and age at onset lower than those of switchers, and as expected, the mean EDSS was also lower. However, their EDSS values of 1.8 and 2.7 are far from the value of 3.5 indicated by Weinshenker as the cut-off beyond which the MS course proceeds, regardless of the past, according to the well-known memoryless theory of the disease [31]. This is an important observation, since the EDSS/age Kaplan–Meier curves diverge significantly, ranging from 0.5 to 3.5 and 0.5 to 6.5 but not falling in the 3.5–6.5 range. These data mean that a smaller number of naive patients reached the cut-off as compared to switchers and, above all, that DMT can significantly modify the MS course but only if started before reaching an EDSS value of 3.5. This also applied to DD.

Based on the graph in Figure 1, these correlations describe an age-related phenomenon that begins at the age 35 and stabilizes at ≥40. These data agree with Confavreux’s findings on the natural history of MS [32].

This author indicated an EDSS value of 4.0 as the disability threshold beyond which the irreversible progression of disability occurs in MS and highlighted the role of age at onset in predicting the time to reach milestones 4.0 and 7.0. Our data are very similar, indicating age as the predicting variable for clinical outcome and DMF response. This interpretation supports the two-stage evidence of disability progression in MS [33] and provides further insight into the mechanisms of action of DMF’s therapeutic effect, particularly in the early phase of disease. This efficacy is expressed, above all, in the naive patients, persisting even in the advanced phase.

The particular performance of DMF, depending on the age and nature of the MS subgroup, is also confirmed by the DNI. In DNI analysis, the 69% difference in mean EDSS values between naive patients and switchers correlated with age (Figure 5) and DD. Notably, this measure rapidly declined with increasing disability, showing an inverse correlation with the same EDSS score (Figure 4).

This means that, at the same age and TinT (paired subjects between groups), the differential disability between naive patients and switchers is high in younger patients and, in particular, in the naive population. This discrepancy in differential disability between naive patients and switchers decreases with age, remaining approximately equal to that of switchers after age 35–40. This differing development in disability accumulation, which reflects the inverse statistical correlation of DNI with EDSS and age is already an established finding. The result is confirmed here with a high level of evidence, functioning as a general law of MS: in a population aged between 18 and 65 years, the greatest response to the drug is obtained at a younger age—in particular, in naive subjects; its effectiveness gradually decreases, starting at age 35 and stabilizing at age 40.

The final confirmation comes from clinical data. The mean ARR_pre_ in naive patients is about twice as high as in switchers, as expected in active MS and in the suboptimal DMT response. However, ARR_post_ was strongly reduced in both groups—mostly in the naive group—according to a trend of significant intergroup differences. Again, this indicates great drug performance at same TinT in younger and naive populations. Moreover, 90% of disease activity occurred in switchers, with a mean age of 41.2 ± 2.1 years, corresponding to an EDSS of 5.3 ± 1.1 and DD of 13.8 ± 3.4. Furthermore, a large proportion of these patients worsened only clinically, with no detectable variation in LL. This condition is expected in the degenerative phase of the disease with disability progression due to prevalent axonal loss. These data confirm the prominent roles of age and the belonging disease subgroup in determining the drug response in MS. Specifically, younger age is related to better DMF performance in terms of preventing disability progression and DNI reduction; on the other hand, higher age is related to poorer effectiveness of DMF in terms of preventing neurological impairment and DNI deterioration.

An important implication of the age-related response to DMT in MS concerns the assessment of the risk-to-benefit ratio for therapeutic decision making in the management of MS, considering the increasing oncological and infectious risk in the age range of 45–50 and the concomitant reduction in drug performance at such ages.

A limitation of the study is the ARR being assessed in the year before and after study entry. It is known that patients with fewer relapses in the year preceding enrollment had a lower ARR during the study period; but above all, the person-year ARR method, by itself, appears unsuitable for detecting the accumulation of disability independent of relapses [34]. Granting that most overall disability in MS is not associated with overt relapses [35], we properly studied it here with the EDSS, in accordance with current literature. The small sample size represents another limitation of this study, potentially compromising the extrapolation of results to a broader clinical setting. However, we believe that the reliability of the study is maintained due to the use of stringent statistical methods. In fact, in terms of descriptive statistics, we applied Leven’s test for mean comparison in order to assess the sample’s homogeneity. In all cases, the method returned high significance and clinical coherence.

Moreover, in inferential statistics, we obtained high levels of significance in the correlation methods of the variables. Finally, the consistency of the Kaplan–Meier curves and the reproducibility of the age/DD graphics with the EDSS-labeled cases suggest good reliability and statistical strength of association.

## 4. Materials and Methods

*Study population*: In a retrospective clinical study, we enrolled all MS patients treated with DMF (Biogen Netherlands B.V., Prins Mauritslaan 13, 1171 LP Badhoevedorp, The Netherlands) afferent at the Multiple Sclerosis Centre of the Neurological Department at the “F. Ferrari” Hospital in Casarano, Lecce (Italy); the “A. Perrino” Hospital in Brindisi (Italy); and the “V. Fazzi” Hospital in Lecce (Italy)—located in the Salento peninsula in the extreme south of Italy. Data collection and coordination of clinical activities were conducted at the “F. Ferrari” Casarano Hospital, which acted as the reference center.

*Patient selection*: From an initial population of 234 DMF-treated patients, we selected 169 MS subjects according to their similar TinT scores, with DMF ranging from 3.7 to 8.8 years. Of these, 74 were naive at the start of treatment and 95 were lateral switchers. A proportion of 29.5% of switcher patients changed therapy due to the ineffectiveness of their previous treatment (interferon and glatiramer acetate), and the remainder changed for safety (increased liver transaminases and recurrent upper respiratory tract infections (from Teriflunomide and Azathioprine) and tolerability (needle intolerance for injectable drugs). Due to the higher efficacy of DMF compared to first-line injectable therapies, the escalation paradigm was avoided and the previous DMT was discontinued.

The inclusion and exclusion criteria were applied as previously described [7]. Briefly, all enrolled patients had a diagnosis of clinically defined MS (McDonald criteria, revised in 2017 by Polman) [20] without limitation of DD; age ranging from 18 to 60 years; EDSS scores ranging from 0.5 to 6.5; baseline MRI of the brain and spinal cord performed no more than three months before study entry; and regular follow-up with quarterly neurological assessment started at least three months before study entry. The exclusion criteria included all metabolic and complex internal comorbidities; all other comorbidities not associated with organ damage were allowed. In addition, we excluded the drop-out subjects. These patients discontinued DMT treatment early due to adverse reactions, poor tolerance to the drug or not reaching the minimum follow-up period of 24 months needed for enrollment. We did not consider patients expressing EDSS ≥ 7.0 since these subjects in the end stage of disease frequently suspend therapy and present difficulties in accessing the hospital.

*Study design*: This was an open-label, clinical and paraclinical multicenter, retrospective and cross-sectional study that started in May 2017 and ended in May 2020. All enrolled MS patients had completed at least three years of treatment with DMF and underwent neurological assessment at study entry, including EDSS calculation, as well as brain and spinal cord MRIs. These paraclinical data and the EDSS itself were compared with those of the pre-study annual follow-up of each patient.

To make the two study populations homogeneous and statistically comparable for investigation purposes, we divided them according to increasing age ranks of five years each, ranging from 18 to 60 years. In this way, each member of a group had a paired subject for comparison, despite any possible mean age differences between members of the comparison groups. The evaluation of increases in EDSS and LL was carried out as previously described [10]. Briefly, 0.5-point increases in the EDSS score and sub-scores detected in two consecutive neurological assessments were considered significant indicators for CDP. Similarly, increasing LL resulting from new T2-weighted or T2-weighted enlarging lesions and the appearance of contrast-enhancing lesions in MRI were considered paraclinical indicators of disease worsening. “MIPAV” software was used to calculate LL (Medical Imaging Processing, Analysis and Visualization, version 7.2.0, National Institutes of Health Center for Information Technology, Rockville, MD, USA).

*Considered variables*: The considered variables, including age, age at onset of disease, DD, TinT and EDSS, were evaluated at the start of patients’ enrollment. In both groups, we also assessed the annual relapse rate (ARR) in the year before the start of DMF (ARR_pre_) and in the year after (ARR_post_). The proportion of paraclinical worsening was also evaluated among progressive patients expressing an EDSS score ≥3.5. The EDSS and MRI assessments were performed by the same qualified operator (RDM) throughout the study to avoid statistical bias between the first and second operator.

*Statistics*: We used version 24.0 of the Statistical Package for Social Science (SPSS 24.0) for descriptive and nonparametric inferential statistics. Specifically, differences between means of considered variables were assessed using the Mann–Whitney U-test (*p* < 0.05), while correlations between them were evaluated using the Spearman rank test. Age-adjusted Kaplan–Meier curves were applied to represent the proportion of patients within the 0.5–3.5 and 3.5–6.5 EDSS intervals over the considered age ranges. Finally, the homogeneity of the distribution of each variable between the comparison groups was assessed using Leven’s test.

## 5. Conclusions

In conclusion, age—especially in the naive subgroup of pathology—represents the major contributor to disability, as well as the main predictive variable of the DMF response in MS. In particular, starting at age 35 and stabilizing at 40, the cut-off range represents the threshold above which patients show increased susceptibility to disability accumulation, irrespective of LL and DMT. In summary, the efficacy of DMF in MS strongly depends on the age and characteristics of patients, being maximal by age 35 and in the naive disease subgroup.

## Figures and Tables

**Table 1:** Demographic and clinical variables of the two study MS populations: naive patients and switchers. ARR_pre_—annual relapse rate in the year before the start of DMF; ARR_post_—annual relapse rate in the year after the start of DMF.

	Naive (*n* = 74) Mean ± SD (95% CI)	Switchers (*n* = 95) Mean ± SD (95% CI)	*p*
**Female-to-male** **sex ratio**	1.9:1.2	1.8:1.1	0.416
**Age** (years)	35.5 ± 10.3 (32.5–38.8)	40.2 ± 8.7 (38.6–41.7)	0.005
**Age at onset** (years)	29.0 ± 5.4 (26.7–31.1)	29.6 ± 4.5 (25.8–30.9)	0.566
**Disease Duration** (years)	5.9 ± 2.7 (3.8–8.4)	11.2 ± 7.1 (10.0–12.5)	<0.001
**Time in Therapy** (years)	5.8 ± 2.1 (3.7–7.9)	6.1 ± 2.5 (3.6–8.8)	0.432
**Expanded Disability Status Scale score**	1.8 ± 1.0 (1.5–2.2)	2.7 ± 1.7 (2.4–3.0)	<0.001
**ARR_pre_**	0.87 ± 0.56 (0.7–1.0)	0.48 ± 0.28 (0.35–0.51)	<0.001
**ARR_post_**	0.01 ± 0.0	0.11 ± 0.0	0.015

## Data Availability

The original contributions presented in this study are included in the article. Further inquiries can be directed to the corresponding author.

## Abbreviations

ARR	annual relapse rate
BBB	blood–brain barrier
CDP	confirmed disability progression
CNS	central nervous system
DD	disease duration
DMF	dimethyl fumarate
DMTs	disease-modifying therapies
DNI	differential neurological impairment
EDSS	expanded disability status scale
LL	lesion load
MS	multiple sclerosis
TinT	time in therapy
